# Effects of drug-induced sleep endoscopy in children with conventional obstructive sleep apnea–hypopnea syndrome: a systematic review and meta-analysis

**DOI:** 10.1007/s11325-023-02945-7

**Published:** 2023-11-02

**Authors:** Xin Wang, Yong-chao Chen, Lan Li, Hong-guang Pan, Yi-shu Teng

**Affiliations:** 1 Department of Otorhinolaryngology, Shenzhen Children’s Hospital, Shenzhen, Guangdong China; 2 Department of Otorhinolaryngology, Shenzhen Children’s Hospital, China Medical University, Shenzhen, Guangdong China

**Keywords:** Drug-induced sleep endoscopy, Obstructive sleep apnea–hypopnea syndrome, Child, Meta analysis, Systematic review

## Abstract

**Purpose:**

This study aimed to conduct a meta-analysis and systematic review of drug-induced sleep endoscopy (DISE) in pediatric conventional obstructive sleep apnea–hypopnea syndrome (OSAHS) without previous upper airway surgery, or comorbidity, to evaluate the change in treatment strategies and to identify obstructive sites observed during DISE. This study aimed to explore the role of DISE in the management of pediatric conventional OSAHS.

**Methods:**

A comprehensive search was conducted using both computerized and manual methods to retrieve relevant case studies on DISE-guided treatment of pediatric conventional OSAHS from databases including PubMed, EMBASE, Cochrane Library, Web of Science, CNKI, WF, and VIP database. The search period extended from database inception to January 2023. Strict inclusion and exclusion criteria were applied to select relevant literature, and data extraction was performed accordingly. Meta-analysis was conducted using the Stata 16.0 software.

**Results:**

A total of 761 patients from four studies were included in the meta-analysis. All pediatric patients had no history of upper airway surgery, craniofacial abnormalities, or syndromes other than OSAHS. The quality assessment revealed that the included studies were of low methodological quality and consisted of non-randomized case studies. Meta-analysis results indicated that in pediatric patients with OSAHS, the obstruction rates observed during DISE were as follows: nasopharyngeal (adenoid) obstruction 93%, soft palate obstruction 35%, oropharyngeal (tonsil) obstruction 76%, tongue base obstruction 32%, supraglottic obstruction 31%, and multi-level obstruction 60%. DISE led to a change in the conventional surgical approach in 45% (95% CI: 29–60%) of patients with OSAHS, providing individualized treatment plans. Postoperative symptoms and sleep-related parameters improved significantly compared to preoperative values, with DISE findings possibly enhancing surgical success rates and potentially avoiding unnecessary procedures.

**Conclusion:**

In some cases, DISE may potentially lead to alterations in conventional surgical approaches for children with OSAHS who had no history of upper airway surgery, craniofacial abnormalities, or other syndromes.. The results of our meta-analysis were in favor of DISE-directed approach for pediatric conventional OSAHS. However, further high-quality randomized controlled trials (RCTs) are warranted in future research to investigate the role of DISE in the management of pediatric OSAHS.

## Introduction

Pediatric obstructive sleep apnea–hypopnea syndrome (OSAHS) is a clinical condition characterized by recurrent partial or complete upper airway obstruction leading to hypoventilation and respiratory pauses during sleep in children. It is relatively common among children, with a prevalence ranging from 1.2 to 5.7% [1, 2]. However, it is generally believed that the prevalence of OSAHS is underestimated [1, 2]. Depending on the severity, OSAHS can have adverse effects on children’s health, including behavioral problems, neurocognitive function, cardiovascular health, endocrine metabolism, and growth and development [1, 3, 4]. Adenotonsillar hypertrophy and tonsillar enlargement are considered the most common causes of pediatric OSAHS in healthy children. Therefore, the American Academy of Pediatrics (AAP) recommends adenotonsillectomy (T&A) as the first-line treatment for pediatric OSAHS [1]. However, research has shown that the occurrence of persistent OSAHS after T&A surgery ranges from 21 to 75% based on postoperative apnea–hypopnea index (AHI) findings [5–8]. A meta-analysis of 1079 pediatric OSAHS indicated that up to 33.7% of children continue to experience sleep breathing disorders after T&A [9]. Apart from systemic diseases and related syndromes, multi-level upper airway narrowing or obstruction is considered a significant factor in treatment-resistant obstructive sleep apnea [10, 11]. Therefore, preoperative assessment of upper airway obstruction levels is crucial to improving the effectiveness of OSAHS treatment.

Drug-induced sleep endoscopy (DISE) is a reliable new technique for assessing the upper airway, which can identify obstructive sites missed during awake examination and provide targeted and effective treatment plans for children, including non-surgical and surgical interventions [12]. In children, most DISE procedures are performed on cases of persistent OSAHS after T&A and special populations, such as those with syndromes or neuromuscular abnormalities, and consensus guidelines have been developed for these scenarios [11, 13–15]. However, there is ongoing debate about whether pediatric OSAHS without previous upper airway surgery or comorbidity are ideal candidates for DISE. In 2015, Galluzzi et al.‘s systematic review suggested that the widespread use of DISE in pediatric patients with OSAHS is not advisable [16]. However, Boudewyns et al. [17] offered a different perspective, stating that DISE aids in decision-making for routine OSAHS surgeries but requires large-sample and RCT studies to prove this conclusion. It is worth noting that Galluzzi et al.’s study included many special populations, which may have influenced the results. In 2017, Gazzaz et al. [18] found that conventional OSAHS without a history of upper airway surgery, craniofacial abnormalities, or syndromes were ideal candidates for DISE. In their study of 558 patients, 35% of children changed their surgical approach based on the findings of DISE. To investigate the impact of DISE on decision-making for conventional OSAHS (no history of upper airway surgery, craniofacial abnormalities, or other syndromes) surgeries, this study conducts a meta-analysis and systematic review by searching all eligible literature on the change in treatment strategies and obstructive sites found during DISE in pediatric conventional OSAHS. This research aims to explore the role of DISE in the management of pediatric conventional OSAHS.

## Patients and methods

### Search strategies

Two authors (YC. C. and X. W.) have conducted comprehensive search in PubMed, EMBASE, Cochrane Library, Web of Science, China National Knowledge Infrastructure (CNKI), WANFANG Data databases (WF), and VIP database (VIP) up to January 31, 2023, that investigate the role of DISE in the treatment of pediatric conventional OSAHS. We used the following search terms: “snoring,” “sleep apnea,” “obstructive sleep apnea hypopnea syndrome,” “OSAHS,” “obstructive sleep apnea,” “OSA,” “drug-induced sleep endoscopy,” “drug-induced sedation endoscopy,” “DISE,” “child,” “children,” “pediatric” separated by the Boolean operator AND or OR. Additionally, manual searching of selected literature was conducted.

### Inclusion and exclusion criteria

The inclusion criteria were (1) original studies published in either Chinese or English; (2) studies involving pediatric participants diagnosed with OSAHS, with no restrictions on gender, ethnicity, geographic origin, disease severity, or duration; (3) children undergoing DISE prior to first-line treatment for OSAHS, with guidance on treatment; (4) the primary outcome is the frequency of treatment plan modifications after DISE, and secondary outcomes include identification of obstructed sites during DISE and treatment-related information.

The exclusion criteria were (1) publications in languages other than Chinese or English; (2) unpublished or incomplete original data, inability to obtain important data after contacting the authors; (3) general reviews or duplicate publications, or in the case of multiple publications targeting the same population, only the highest-quality or largest sample size publication was selected; (4) unclear diagnostic criteria, a history of upper airway surgeries such as adenotonsillectomy, or comorbidities such as Down syndrome, severe craniofacial anomalies, or neuromuscular diseases.

### Study selection and data extraction

Two authors (YC. C. and X. W.) identified and screened potentially eligible studies by reviewing the title and abstract of each article. Relevant publications were then selected for full-text review. Studies not clear were discussed and decided whether excluding it. In the case of discrepancies in included study between investigators, a third investigator (YS. T.) made the definitive decision via discussion.

YC. C. and X. W., respectively, assessed the relevance of search results and extracted the basic information of included study in Excel format. Discrepancies were resolved by discussion that consisted until consensus was achieved. Data extraction included general information (first author, publication year, study design, sample size), demographic data (age, gender, BMI, etc.), intervention details (medications used in DISE), and outcome data (frequency of treatment plan modifications, obstructed sites identified during examination, treatment details). Data extraction followed the methods outlined in the Cochrane Handbook for Systematic Reviews [19].

### Quality assessment of included studies

The Newcastle–Ottawa Scale (NOS) [20] was used to assess the quality of included studies. Criteria assessed included selection (0–4 points), comparability (0–2 points), and outcome/exposure (0–3 points).

### Statistical analysis

Single-arm meta-analysis was conducted using Stata 17.0. Heterogeneity was assessed using the *Q*-test and *I*^2^-statistic. If *P* ≥ 0.10 and *I*^2^ ≤ 50.0%, indicating homogeneity among studies, a fixed-effects model was employed. Otherwise, a random-effects model was used, and subgroup and sensitivity analyses were performed to explore potential sources of heterogeneity (e.g., sample size). A 95% confidence interval (CI) was used for interval estimation, with a significance level of *α* = 0.05.

## Results

### Literature screening results

**Fig. 1:**
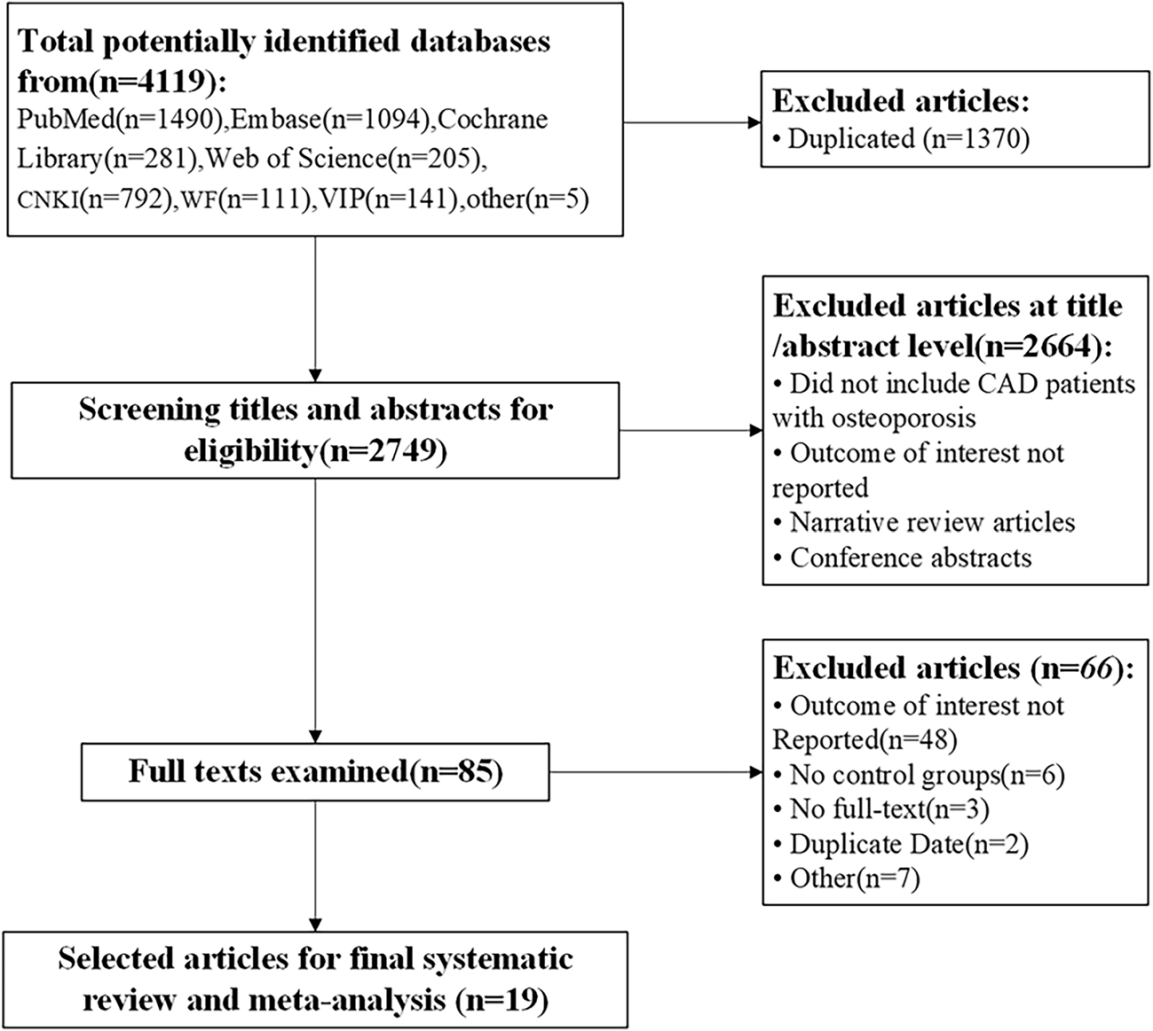
Flowchart showing the selection of studying for the meta-analysis

Following the retrieval strategy, a total of 425 articles were retrieved, distributed among various databases as follows: 146 from PubMed, 183 from Embase, 146 from Cochrane Library, 160 from Web of Science, 100 from CNKI, 131 from WF, and 110 from VIP. After deduplication and screening of titles, abstracts, and full texts, a strict adherence to the inclusion and exclusion criteria resulted in the final inclusion of 4 studies [10, 18, 21, 22], comprising a total of 761 pediatric cases. The literature screening process and outcomes are illustrated in Fig. 1. Notably, two publications by Chen et al. [21, 23] pertained to the same study population, and both were considered in the data analysis. Table 1 presents general information about the included studies and their quality assessment. The quality assessment revealed that the methodological quality of the included studies was generally low, as all were non-randomized case studies.

**Table 1:** Description of characteristics of eligible studies

First author[ref] (Year)/Country	Study design	Sample Size T/M/F	Age (y)	BMI (kg/m^2^)	Single-Level/Multi-Level Obstruction	Medication Used in DISE	Severity of OSAHS	Outcome	Follow-up	NOS
Boudewyns [11] (2014)/Belgium	Prospective	37/10/27	4.1 ± 0.9	15.7 ± 0.2	16/21	Isoflurane and propofol	Moderate to Severe OSAHS (OAHI ≥ 2/h)	24.3% changed their treatment plan; OAHI, average SaO_2_, and minimum SaO_2_ improved significantly	NA	6
Gazzaz [18] (2017)/Canada	Retrospective	558/327/231	6.2 ± 2.7	NA	NA	Remifentanil and propofol	NA	35% changed their treatment plan	NA	6
Chen [21, 23] (2019)/China	Retrospective	126/84/42	5.7 ± 3.2	15.7 ± 5.5	NA	Dexmedetomidine	NA	45.2% changed their treatment plan; RDI and minimum SaO_2_ improved significantly compared to before	1 year	6
Williamson [10] (2022)/America	Retrospective	40/29/11	5.0 ± 4.6	69.6 ± 29.7^*^	15/25	Propofol	AHI < 5: 24; AHI 5 ~ 10: 14; AHI > 10: 2	75% changed their treatment plan; symptoms significantly improved; AHI and minimum SaO_2_ improved significantly	At least follow-up for 3 weeks	6

*F* female, *M* male, *NOS* the Newcastle–Ottawa score, *T* total

^*^BMI percentile

### Meta-analysis results 

#### Obstructed sites

**Fig. 2:**
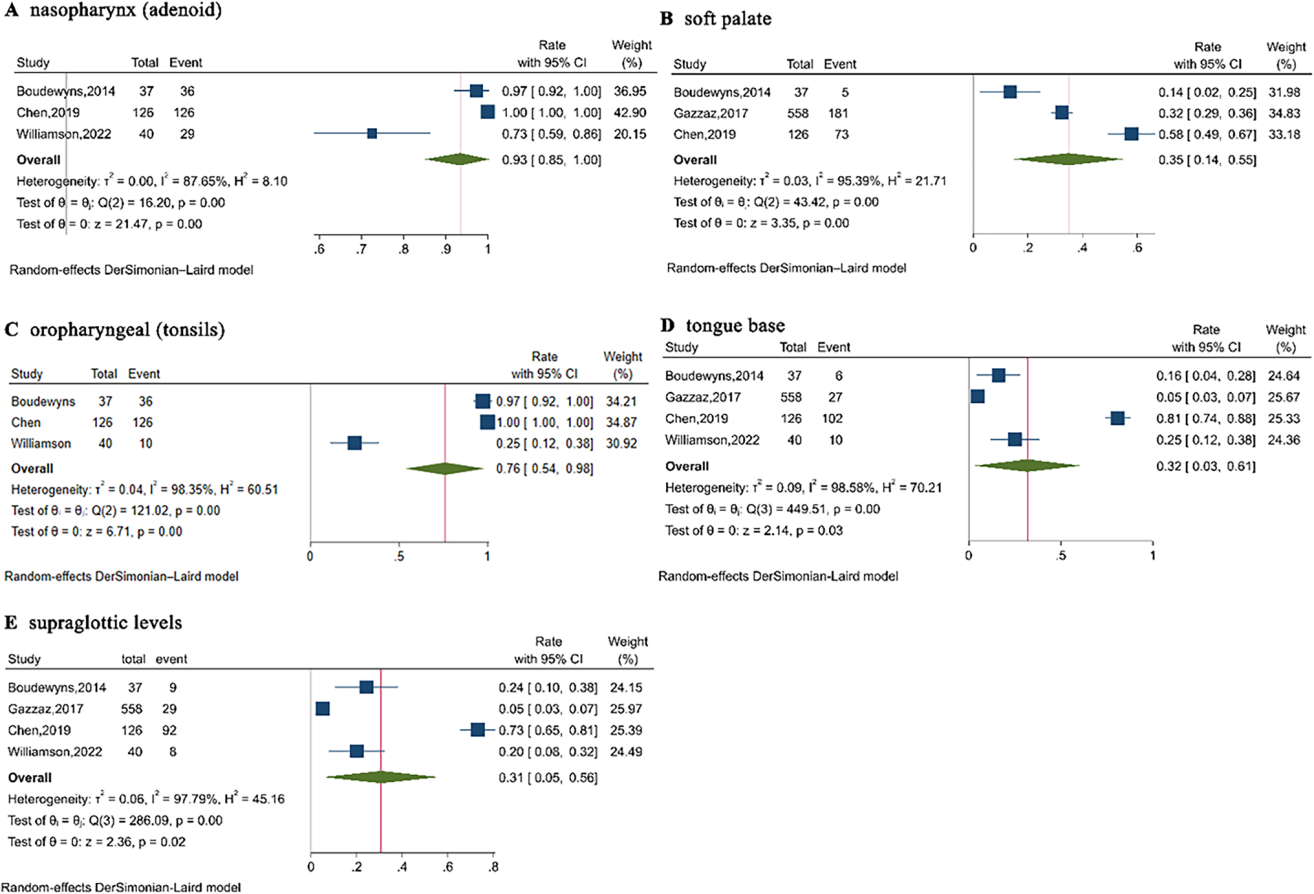
The forest plot of obstruction sites identified by DISE

All four studies reported on the obstructed sites identified during DISE in pediatric conventional OSAHS. However, the study by Gazzaz et al. [18] did not report on nasal and pharyngeal (adenoid) obstruction, while the study by Williamson et al. [10] did not report on soft palate obstruction. Based on the available data, a meta-analysis was conducted on the obstruction rates in the nasopharynx (adenoid), soft palate, oropharyngeal (tonsils), tongue base, and supraglottic levels. Due to heterogeneity among the studies, a random-effects model was used for all analyses. The forest plot (Fig. 2) illustrates the obstruction rates found during DISE: nasopharyngeal (adenoid) obstruction rate of 93% (95% CI: 85–100%), soft palate obstruction rate of 35% (95% CI: 14–55%), oropharyngeal (tonsil) obstruction rate of 76% (95% CI: 54–98%), tongue base obstruction rate of 32% (95% CI: 3–61%), and supraglottic obstruction rate of 31% (95% CI: 5–56%). Sensitivity analyses for each level showed that the exclusion of any single study did not lead to Rate values outside the 95% CI of the overall rate, indicating the robustness of the results.

**Fig. 3:**
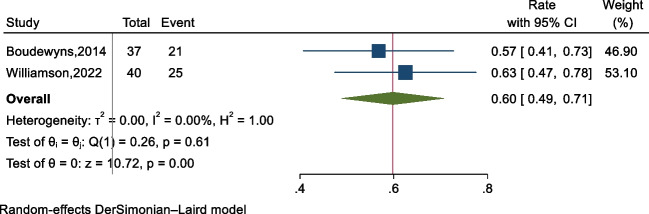
The forest plot of multi-level obstructions identified by DISE

Two studies [10, 11] provided information on multi-level obstruction (defined as obstruction in more than one level, including but not limited to adenoid and tonsil obstruction) in pediatric conventional OSAHS, involving a total of 77 cases. Since the heterogeneity test indicated clinical and statistical homogeneity among the studies (*P* = 0.61, *I*^2^ = 0.0%), a fixed-effects model was used. The forest plot (Fig. 3) shows a multi-level obstruction rate of 60% (95% CI: 49–71%).

#### Rate of treatment modification

**Fig.4:**
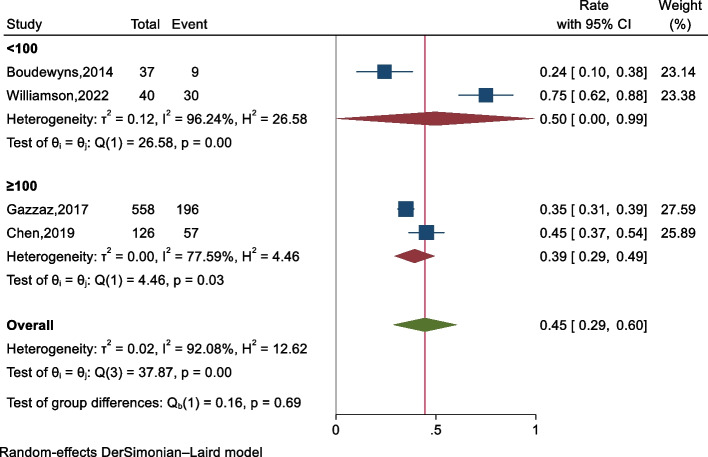
The forest plot of treatment modification rate guided by DISE

**Fig. 5:**
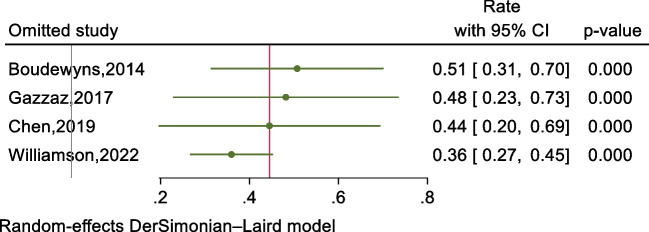
The sensitivity analysis of treatment modification rate guided by DISE

A meta-analysis was conducted on the rate of treatment modification guided by drug-induced sleep endoscopy (DISE) in the 761 patients from the four studies. Due to significant heterogeneity among the studies (*P* = 0.000, *I*^2^ = 92.1%), a random-effects model was utilized. The forest plot (Fig. 4) shows that DISE resulted in treatment modification in 45% (95% CI: 29–60%) of pediatric conventional OSAHS. Subgroup analysis indicated that sample size and the use of medications during DISE had no impact on reducing heterogeneity. Sensitivity analysis (Fig. 5) demonstrated that excluding any single study did not result in rate values outside the 95% CI of the overall rate, reaffirming the reliability of the results.

Three studies provided specific descriptions of the treatment received by patients after DISE (Table 2). Adenoid and tonsillectomy were the primary treatment modalities for pediatric conventional OSAHS. However, some pediatric conventional OSAHS also had indications for tongue base surgery and supraglottic surgery after DISE.

**Table 2:** DISE-guided treatment in the study

Study	Adenoidectomy	Tonsillectomy	Surgery at the level of the tongue base	Supraglottoplasty	Non-surgical treatment
Boudewyns [11]	31 (83.8%)	30 (81.1%)	-	-	4 (10.8%)
Gazzaz [18]	499 (89.4%)	412 (73.9%)	5 (0.9%)	-	9 (18.0%)
Williamson [10]	27 (67.5%)	11 (27.5%)	9 (22.5%)	5 (12.5%)	5 (12.5%)

## Discussion

In recent years, DISE has garnered increasing attention and rapidly evolved as a novel technique for upper airway assessment. To date, there is a wide divergence of opinions regarding the indications for pediatric DISE. Nonetheless, the majority of medical professionals deem DISE suitable for persistent OSAHS after T&A and special populations, such as those with syndromes or neuromuscular abnormalities, and consensus guidelines have been developed for these scenarios [11, 13–15]. However, considerable controversy persists regarding the applicability of preoperative DISE for pediatric conventional OSAHS.

In this study, we employed a meta-analysis and systematic review approach to investigate the role of DISE in the treatment of pediatric conventional OSAHS. A total of four case studies were included, encompassing 761 study subjects who had no history of upper airway surgery, craniofacial anomalies, or syndromes. The quality assessment of the included studies indicated a relatively low methodological quality, as all were non-randomized case studies.

DISE serves as an effective diagnostic tool, capable of identifying obstructive sites that may be overlooked during routine ear, nose, and throat examinations of awake children. Furthermore, it aids in tailoring specific and effective treatment plans for children, thus minimizing the need for unnecessary surgeries [12]. Our findings within the cohort of routine OSAHS in children undergoing DISE revealed obstruction rates in various regions as follows: nasopharynx (adenoids) 93%, soft palate 35%, oropharynx (tonsils) 76%, base of tongue 32%, supraglottis 31%, and multi-level obstruction 60%. A systematic review conducted in 2016 demonstrated that at least one obstructive site was identified in 100% of post-tonsillectomy and adenoidectomy (T&A) persistent OSAHS children who underwent DISE evaluation. The base of the tongue was the most frequently obstructed site, with tongue base tonsillectomy and supraglottoplasty being the most commonly employed treatment methods [24]. These results emphasize the importance of assessing the impact of the soft palate, base of tongue, and supraglottis when evaluating obstruction levels in pediatric conventional OSAHS.

Our meta-analysis results indicate that DISE altered the conventional surgical approach in 45% (95% CI: 29–60%) of pediatric conventional OSAHS. Within the included studies, DISE provided personalized treatment plans for children, leading to significant improvements in postoperative symptoms and sleep-related parameters compared to preoperative conditions. This substantially increased the surgical success rate while avoiding unnecessary surgeries. Therefore, preoperative DISE in pediatric conventional OSAHS can effectively identify upper airway obstructions, reducing the likelihood of postoperative persistent OSAHS and the need for secondary surgeries. Boudewyns et al. [11] reported a surgical success rate of 91% in 22 patients with PSG data guided by DISE. Chen et al.‘s study demonstrated significant improvements in preoperative and postoperative RDI and lowest SaO_2_. Williamson et al.‘s research indicated that under DISE-guided treatment, symptoms significantly improved in 91.4% of children, with significant improvements in AHI and lowest SaO_2_ observed in 13 patients with PSG data [10].

The results of our meta-analysis were in favor of DISE-directed approach for pediatric OSAHS without previous upper airway surgery or comorbidity. In the context of expanding indications, DISE should be considered in these patient population. It is worth acknowledging that the good effect of T&A, and T&A attains a success rate within the range of 71 to 87% in healthy children with OSAHS [6, 25]. Nevertheless, it is crucial to recognize that factors such as obesity, small tonsil size, and severe AHI independently pose increased risks for postoperative persistence of OSAHS in children [10, 26]. In the presence of these independent risk factors for ongoing disease, DISE-directed approach should rather be recommended for them.

Currently, anesthesia medications that can be used for pediatric DISE, including propofol, dexmedetomidine, and midazolam, but each exhibiting distinct effects on respiratory physiology [14, 27–29]. Dexmedetomidine possesses sedative and analgesic properties while exerting minimal influence on respiratory depression [30, 31]. However, the use of propofol in DISE presents complexities due to its potential to induce muscle relaxation, decrease ventilatory drive, and lead to respiratory depression, particularly in patients with OSAHS [30, 31]. Among the included studies, pediatric patients undergoing DISE are typically sedated using either a continuous intravenous infusion of propofol or dexmedetomidine as the most common sedation options. The variability in obstruction patterns observed with different DISE medications is a consideration in the surgical decision-making process. In the context of adult DISE, certain comparative studies between dexmedetomidine and propofol have reported a higher incidence of respiratory depression and tongue base obstruction associated with propofol [32]. However, in accordance with the research findings presented by Kirkham et al. [33], there was no statistically significant difference in the likelihood of ≥ 50% obstruction during DISE when utilizing either dexmedetomidine or propofol at various anatomical levels within a cohort of 117 pediatric patients diagnosed with OSAHS. Similarly, we conducted subgroup analyses on the basis of medication status and that had no impact on reducing heterogeneity. Hence, we contend that minor variances in DISE medications would likely exert limited clinical repercussions in practice.

Several studies have also affirmed the role of DISE in the treatment of pediatric conventional OSAHS [13, 34–38]. However, some of these studies combined pediatric conventional OSAHS with postoperative persistent OSAHS and did not exclude patients with syndromes, resulting in significant heterogeneity among the study subjects. Therefore, they were not included in the meta-analysis. Galluzzi et al. [16] conducted a systematic review of the proportion of tonsillar and/or adenoid hypertrophy in children with OSAHS undergoing DISE, including five studies (*n* = 39). They estimated that tonsillar and/or adenoid hypertrophy accounted for OSAHS in 71% (95% CI: 64–77%) of cases and argued against the widespread use of DISE in children with OSAHS. However, their study suffered from substantial subject heterogeneity (including a significant number of syndrome patients) and did not compare the outcomes of surgeries guided by DISE with those guided by conventional surgical indications. Additionally, they did not provide a comparison of obstructive sites identified by DISE. Therefore, the conclusions of their study are questionable.

Limitations of this study should be acknowledged. First, the inclusion criteria were limited to studies published in Chinese and English, potentially resulting in the omission of relevant literature published in other languages, which could lead to incomplete coverage of the available literature. Second, the clinical evidence level within the included studies is relatively low, primarily due to the absence of high-quality, rigorously conducted randomized controlled trials (RCTs) incorporating blind methodologies. Due to the absence of randomized controls, our conclusions regarding DISE’s impact on outcomes and the avoidance of unnecessary procedures are speculative. Third, the scarcity of detailed information in the included studies hindered the ability to perform subgroup analyses based on factors such as BMI or tonsil size, potentially introducing significant clinical heterogeneity.

## Conclusions

In some cases, DISE may potentially lead to alterations in conventional surgical approaches for children with OSAHS who had no history of upper airway surgery, craniofacial abnormalities, or other syndromes. The results of our meta-analysis were in favor of a DISE-directed approach for pediatric conventional OSAHS. However, further high-quality randomized controlled trials (RCTs) are warranted in future research to investigate the role of DISE in the management of pediatric OSAHS.

## Data Availability

The datasets used and/or analyzed during the current study are available from the corresponding author on reasonable request.

## Declarations

### Additional information

This article does not contain any studies with human participants performed by any of the authors.
